# Nuclear factor Nrf2 promotes glycosidase OGG1 expression by activating the AKT pathway to enhance leukemia cell resistance to cytarabine

**DOI:** 10.1016/j.jbc.2022.102798

**Published:** 2022-12-14

**Authors:** Qin Shang, Chengyun Pan, Xi Zhang, Tonghua Yang, Tianzhen Hu, Lin Zheng, Shuyun Cao, Cheng Feng, Xiuying Hu, Xiao Chai, Jishi Wang, Qin Fang

**Affiliations:** 1 College of Pharmacy, Guizhou Medical University, Guiyang, Guizhou, China; 2 Department of Haematology, Laboratory of Hematopoietic Stem Cell Transplantation Centre of Guizhou Province, Guiyang, Guizhou, China; 3 Department of Haematology, Affiliated Hospital of Guizhou Medical University, Guiyang, Guizhou, China; 4 Medical Center of Hematology, The Xinqiao Hospital of Third Military Medical University, Chongqing, China; 5 Department of Hematology, Yunnan Blood Disease Clinical Medical Center, Yunnan Blood Disease Hospital, National Key Clinical Specialty of Hematology, The First People’s Hospital of Yunnan Province, Kunming, China; 6 Department of Clinical Medical School, Guizhou Medical University, Guiyang, Guizhou, China; 7 National Clinical Research Center for Hematologic Diseases, The First Affiliated Hospital of Soochow University, Suzhou, China; 8 Department of Pharmacy, Affiliated Hospital of Guizhou Medical University, Guiyang, Guizhou, China

**Keywords:** acute myeloid leukemia, cytarabine, 8-hydroxyguanine DNA glycosidase, nuclear factor E2–related factor 2, resistance, AML, acute myeloid leukemia, Ara-C, cytarabine, BER, base excision repair, BM, bone marrow, ChIP, chromatin immunoprecipitation, CON, control, CT, cycle threshold, DDR, DNA damage repair, EV, empty vector, FCM, flow cytometry, ICC, immunocytochemistry, IF, immunofluorescence, IHC, immunohistochemistry, NC, negative control, NOD/SCID, nonobese diabetic/severe combined immunodeficiency, Nrf2, nuclear factor E2–related factor 2, OGG1, 8-hydroxyguanine DNA glycosidase, 8-OHdG, 8-hydroxy-2-deoxyguanosine, p-AKT, phosphorylated AKT, qPCR, quantitative PCR, si-Nrf2, silenced Nrf2, si-OGG1, OGG1 siRNA

## Abstract

Chemotherapy resistance is the dominant challenge in the treatment of acute myeloid leukemia (AML). Nuclear factor E2–related factor 2 (Nrf2) exerts a vital function in drug resistance of many tumors. Nevertheless, the potential molecular mechanism of Nrf2 regulating the base excision repair pathway that mediates AML chemotherapy resistance remains unclear. Here, in clinical samples, we found that the high expression of Nrf2 and base excision repair pathway gene encoding 8-hydroxyguanine DNA glycosidase (OGG1) was associated with AML disease progression. *In vitro*, Nrf2 and OGG1 were highly expressed in drug-resistant leukemia cells. Upregulation of Nrf2 in leukemia cells by lentivirus transfection could decrease the sensitivity of leukemia cells to cytarabine, whereas downregulation of Nrf2 in drug-resistant cells could enhance leukemia cell chemosensitivity. Meanwhile, we found that Nrf2 could positively regulate OGG1 expression in leukemia cells. Our chromatin immunoprecipitation assay revealed that Nrf2 could bind to the promoter of OGG1. Furthermore, the use of OGG1 inhibitor TH5487 could partially reverse the inhibitory effect of upregulated Nrf2 on leukemia cell apoptosis. *In vivo*, downregulation of Nrf2 could increase the sensitivity of leukemia cell to cytarabine and decrease OGG1 expression. Mechanistically, Nrf2–OGG1 axis–mediated AML resistance might be achieved by activating the AKT signaling pathway to regulate downstream apoptotic proteins. Thus, this study reveals a novel mechanism of Nrf2-promoting drug resistance in leukemia, which may provide a potential therapeutic target for the treatment of drug-resistant/refractory leukemia.

Acute myeloid leukemia (AML) refers to a malignant tumor with uncontrolled proliferation of immature myeloid cells. The standard induction chemotherapy for AML is “3 + 7” regimen, that is, 3-day anthracycline + 7-day cytarabine (Ara-C) for remission. After chemotherapeutic treatment, only a few AML patients survived for over 5 years, and most patients died of relapse or some related complications. Therefore, chemotherapy resistance has turned into a leading challenge in AML treatment.

Studies have proved that AML has varieties of drug resistance mechanisms, including adaptive cytoprotection mechanism (1), tumor microenvironment protection (2, 3), autophagy (4), and epigenetic mutation (5). In addition, chemotherapy often accompany with DNA damage. Once intracellular DNA is damaged, the original structure can be restored and DNA damage repair (DDR) can be completed under the action of enzymes. DDR pathway mainly includes base excision repair (BER), homologous recombination, nucleotide excision repair, mismatch repair, as well as nonhomologous end joining (6). There exists a certain relationship between DDR pathway and drug resistance (7). In the DDR pathway, BER is responsible for repairing most DNA damage and exerts a vital role in the maintenance of gene integrity (8), which is tightly associated with tumor relapse and drug resistance (9). Therefore, investigating the molecular mechanism of BER pathway and drug resistance is a crucial strategy to overcome clinical relapse and chemotherapy resistance.

Nuclear factor E2–related factor 2 (Nrf2, also known as NFE2L2) is a key factor in antioxidant stress system. Its deletion or activation will directly influence the balance of intracellular redox (10). Based on normal physiological situations, Nrf2 binds to Kelch-like ECH-related protein 1 (Keap1), existing inactively in the cytoplasm. Nevertheless, the ability of Keap1 to ubiquitinate and degrade Nrf2 decreases, resulting in the translocation of Nrf2 to the nucleus to form a heterodimer with small Maf, which binds to antioxidant stress elements to regulate downstream target genes and enhance cytoprotection (11). Nrf2 is abnormally high denoted in multiple cancers including leukemia (12, 13, 14, 15, 16). Nrf2 activation can also promote tumor cell proliferation, inhibit oxidative stress, and promote immune escape, resulting in chemotherapy resistance (17). In melanoma, the inhibition of Nrf2 is an effective strategy to overcome chemotherapy resistance (18). The aforementioned evidence shows that Nrf2 exerts a vital role in cancer progression and chemotherapy resistance.

The most common lesions of DNA oxidative damage are 8-oxo-7,8-dihydro-2′-deoxyguanosine and 8-hydroxy-2-deoxyguanosine (8-OHdG), and there is mutual transformation between them (19). BER pathway generates a leading role in protecting cells in the process of DNA oxidative damage (20), which contains the following enzymes: 8-hydroxyguanine DNA glycosidase (OGG1), AP site endonuclease 1 (APE1), DNA polymerase β (POL-β), poly(ADP-ribose) polymerase 1 (PARP1), and DNA ligase (DNA ligase III/XRCC1) (21). In the BER pathway, OGG1 is an important biomarker, which is mainly responsible for identifying and removing 8-oxo-7,8-dihydro-2′-deoxyguanosine/8-OHdG, so as to maintain the integrity of genome function (22, 23). OGG1 is necessary to maintain the proliferation of tumor cells, tightly involving in the occurrence and development of multiple cancers (24, 25). In leukemia patients, those with high OGG1 expression has a poor prognosis and a higher risk of relapse (26). The oxidative damage of cells treated with silver nanoparticles is achieved by decreasing the nuclear expression of Nrf2 and downregulating OGG1 expression of BER pathway gene (27). In addition, it has been reported that the inhibition of oxidative DNA damage by antioxidants in estrogen-induced breast cancer is related to the upregulation of OGG1 expression by Nrf2 (28). However, it has not been reported whether Nrf2 is involved in regulating BER pathway genes leading to drug resistance in AML.

The current work attempted to investigate the function of Nrf2 and BER pathway rate-limiting enzyme OGG1 in AML resistance. Nrf2 overexpression promoted the expression of OGG1, which then mediated the resistance of AML cells to Ara-C. Based on our findings, the overexpression of Nrf2 could promote the expression of OGG1 through regulating protein kinase B (AKT) signaling.

## Results

### High expression of Nrf2 is associated with the relapse of AML

**Figure 1:**
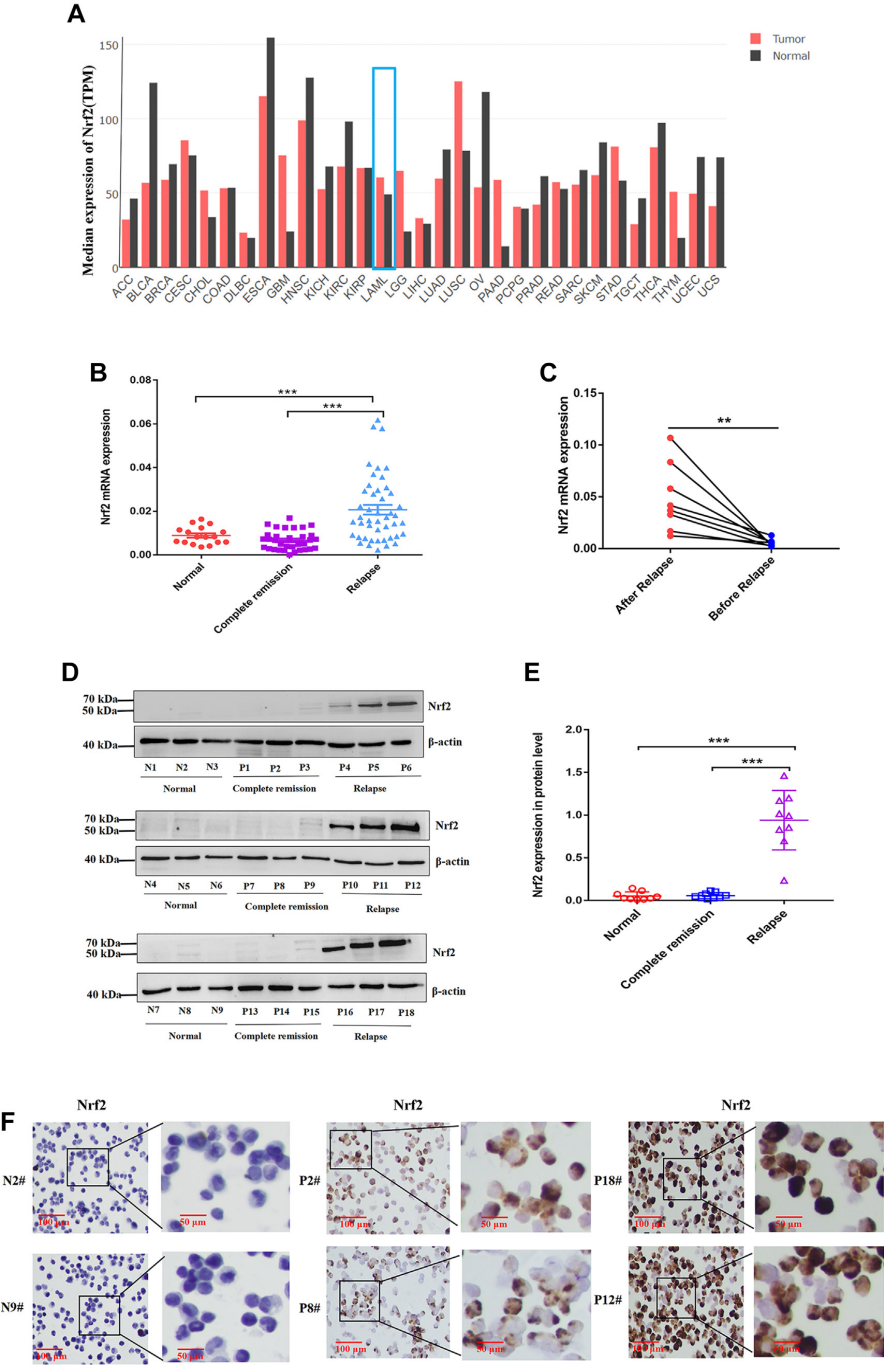
**High expression of Nrf2 associates with AML relapse.***A*, analysis of gene expression profile of Nrf2 in AML samples (n = 173) and matched normal samples (n = 70) according to GEPIA website. *B*, the expression level of Nrf2 mRNA in normal (n = 17), complete remission (n = 33), and relapsed AML patients (n = 46) was identified by RT–PCR. *C*, the expression of Nrf2 was detected by RT–PCR in the same AML patients before and after relapse (n = 8). *D*, Western blot was adopted for detecting the expression level of Nrf2 in normal (n = 9), complete remission (n = 9), and relapsed (n = 9) AML samples. *E*, the relative gray values of Nrf2 expression in clinical samples. *F*, ICC staining was applied to identify the expression of Nrf2 in clinical samples (N2 and N9, normal group; P2 and P8, complete remission group; and P18 and P12, relapse group) (N: normal; P: patient). The scale bars from *left* to *right* represent 100 and 50 μm, respectively. ∗*p* < 0.05, ∗∗*p* < 0.01, and ∗∗∗*p* < 0.001. AML, acute myeloid leukemia; ICC, immunocytochemistry; Nrf2, nuclear factor E2–related factor 2; TPM, transcripts per million.

We first employed GEPIA website to comprehensively explore the expression profiles of Nrf2 in different cancer types. In Figure 1*A*, Nrf2 expression in AML samples was higher than matched normal samples. Nrf2 expressions were then detected in bone marrow (BM) mononuclear cells of normal healthy donors, complete remission, and relapsed AML patients. As presented in Figure 1*B*, Nrf2 mRNA expression in relapsed patients (n = 46) was obviously higher than that in normal healthy donors (n = 17) and complete remission patients (n = 33) (*p* < 0.001). In the same AML patients, Nrf2 mRNA expression after relapse was notably higher than that before relapse (Fig. 1*C*). Western blot also demonstrated that Nrf2 expression in relapsed AML was higher than that in normal healthy donors and complete remission groups (*p* < 0.001, Fig. 1, *D* and *E*). Immunocytochemistry (ICC) staining revealed that Nrf2 expression was higher in relapsed AML patients (Fig. 1*F*). The aforementioned results suggested that high Nrf2 expression could be closely related to AML relapse.

### High OGG1 expression is associated with increased Nrf2 expression in AML

**Figure 2:**
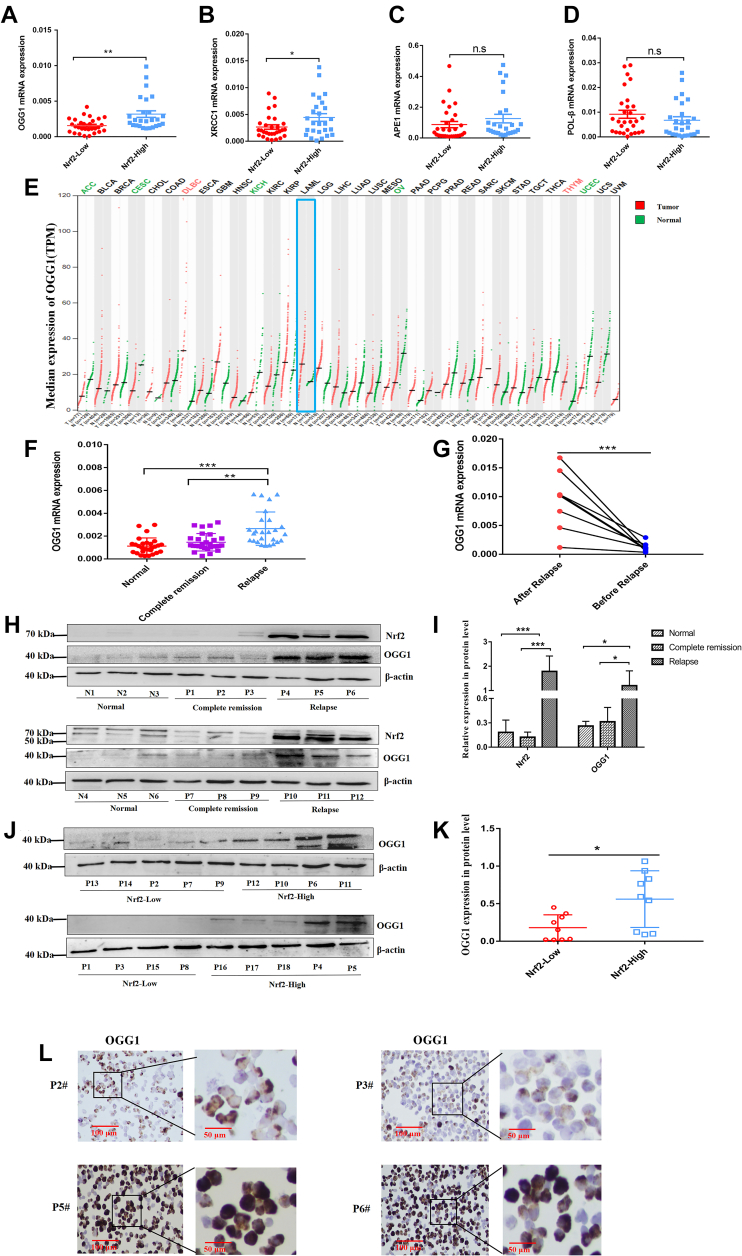
**High expression of OGG1 associates with high expression of Nrf2 in AML.***A*–*D*, RT–PCR analysis of the expressions of OGG1, XRCC1, AP site endonuclease 1 (APE1), and DNA polymerase β (POL-β) in Nrf2-low (n = 29) and Nrf2-high (n = 26) groups. *E*, the gene expression profiles of OGG1 in AML samples (n = 173) and matched normal samples (n = 70) were analyzed according to GEPIA website. *F*, RT–PCR analysis of OGG1 expression in normal (n = 28), complete remission (n = 29), and relapsed (n = 26) AML patients. *G*, the expression of OGG1 in the same AML patients before and after relapse was detected by RT–PCR. *H*, Western blot was employed to detect the expression level of OGG1 and Nrf2 proteins in normal (n = 6), complete remission (n = 6), and relapsed AML patient (n = 6) samples. *I*, the relative gray values of OGG1 and Nrf2 expressions in clinical samples. *J*, Western blot was applied to detect the protein expression of OGG1 in Nrf2-high (n = 9) and Nrf2-low (n = 9) groups. *K*, the relative gray values of OGG1 expression in Nrf2-high and Nrf2-low groups. *L*, ICC staining was used to identify the expression level of OGG1 in clinical samples (P2 and P3, Nrf2-low group; P5 and P6, Nrf2-high group). The scale bars from *left* to *right* represent 100 and 50 μm, respectively. ∗*p* < 0.05, ∗∗*p* < 0.01, and ∗∗∗*p* < 0.001. AML, acute myeloid leukemia; ICC, immunocytochemistry; Nrf2, nuclear factor E2–related factor 2; OGG1, 8-hydroxyguanine DNA glycosidase; TPM, transcripts per million.

To furthermore explore the association of Nrf2 with the crucial pathway mediating drug resistance in AML, we first divided AML samples into Nrf2 high expression (Nrf2-high) group and low expression (Nrf2-low) group based on the median value of Nrf2 expression and compared BER signaling pathway–related factors. Among BER pathway genes, OGG1 and XRCC1 were positively correlated with Nrf2 expression (Fig. 2, *A*–*D*). Through the GEPIA website, we found that the expression of OGG1 in AML samples was higher than that in matched normal ones (Fig. 2*E*). By RT–PCR, we detected OGG1 expression in normal healthy donors (n = 28), complete remission (n = 29), and relapse patients (n = 26), showing notably higher OGG1 expression in relapsed AML patients than that in normal healthy donors and patients with complete remission (*p* < 0.01, Fig. 2*F*). We further investigated OGG1 expression before and after relapse in the same AML patients, finding that OGG1 expression after relapse was obviously higher than that before relapse (*p* < 0.001, Fig. 2*G*). Western blot also demonstrated that OGG1 expression was higher in patients suffering from relapsed AML (*p* < 0.05, Fig. 2, *H* and *I*). In addition, Western blot revealed that OGG1 expression was enhanced in Nrf2-high group compared with Nrf2-low group (*p* < 0.05, Fig. 2, *J* and *K*). ICC staining also indicated that OGG1 expression in Nrf2-high group was higher than that in Nrf2-low group (Fig. 2*L*). The aforementioned results suggested the positive correlation of OGG1 expression with AML relapse and Nrf2 expression.

### High expression of Nrf2 in drug-resistant AML cell lines

**Figure 3:**
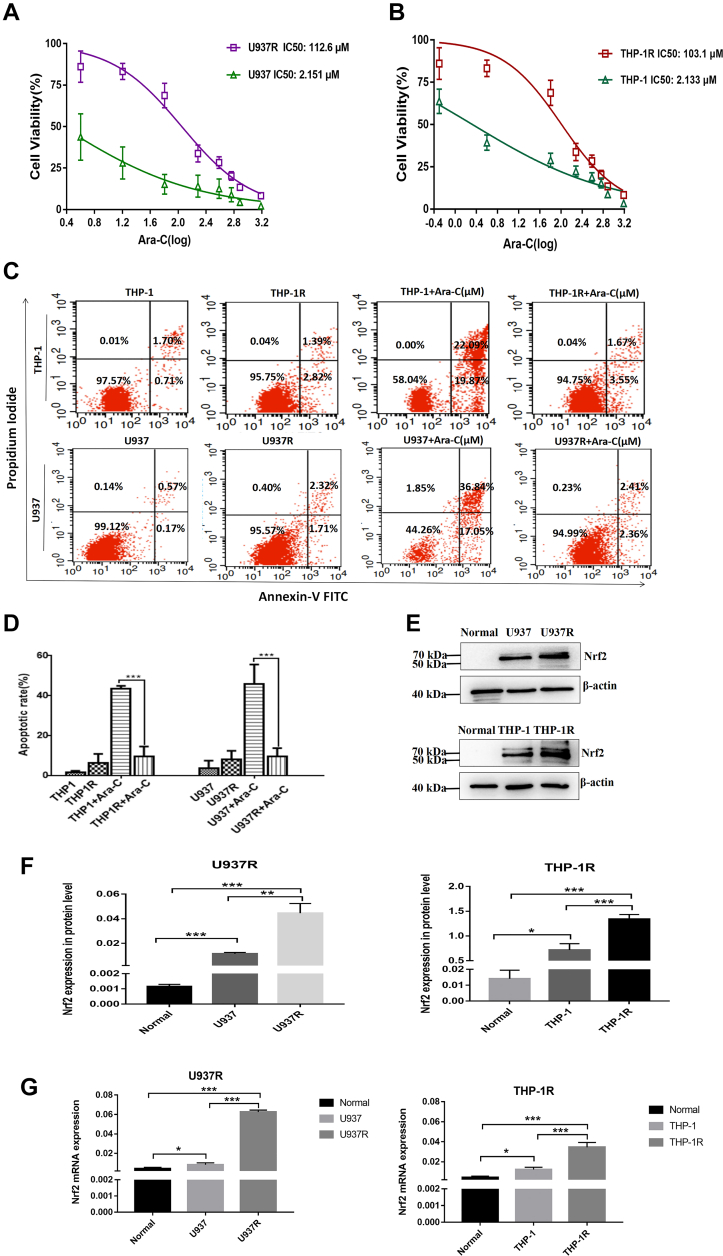
**High expression of Nrf2 in Ara-C-resistant AML cell lines.***A*, U937 and U937R cells were treated with different concentrations of Ara-C (4, 16, 64, 192, 386, 578, 768, and 1536 μM) for 24 h, and the cell viability was detected by CCK-8 colorimetry. *B*, THP-1 and THP-1R cells were treated with different concentrations of Ara-C (0.5, 4, 64, 192, 386, 578, 768, and 1536 μM) for 24 h, and the cell viability was detected by CCK-8 colorimetry. *C*, after U937, U937R, THP-1, and THP-1R were cocultured with Ara-C (2 μM) for 24 h, the apoptosis rate of AML cell lines was detected by FCM. *D*, analysis of apoptosis rate. *E*, Western blot was adopted to detect the expression of Nrf2 in normal (bone marrow mononuclear cells from normal healthy donors) and sensitive AML cell lines (U937 and THP-1) and Ara-C-resistant AML cell lines (U937R and THP-1R). *F*, the relative gray values of Nrf2 expression. *G*, the mRNA expression of Nrf2 in normal, sensitive AML cell lines (U937 and THP-1), and Ara-C-resistant AML cell lines (U937R and THP-1R) was detected by RT–PCR. Each experiment was repeated three times independently and expressed as mean ± SD. ∗*p* < 0.05, ∗∗*p* < 0.01, and ∗∗∗*p* < 0.001. AML, acute myeloid leukemia; Ara-C, cytarabine; CCK-8, Cell Counting Kit-8; FCM, flow cytometry; Nrf2, nuclear factor E2–related factor 2.

At the cellular level, we verified the relationship between Nrf2 and AML resistance. First, we constructed AML-resistant cell lines (U937R and THP-1R) by the method of increasing Ara-C concentrations. To verify the changes of biological characteristics of AML drug–resistant cell lines, we detected the cell viability of AML-sensitive and AML drug–resistant cell lines. The IC_50_ values of U937R and U937 cells subjected to different concentrations of Ara-C were 112.6 and 2.151 μM, respectively, indicating that U937R cells were 52.3 times more resistant to Ara-C than U937 cells (Fig. 3*A*). The IC_50_ values of THP-1R and THP-1 cells were 103.1 and 2.133 μM, respectively, indicating that THP-1R cells were 48.3 times more resistant than THP-1 cells (Fig. 3*B*). Flow cytometry (FCM; BD Biosciences) results also found that the apoptotic numbers of U937 and THP-1 treated with the same concentration of Ara-C for 24 h were significantly higher than those of U937R and THP-1R (Fig. 3, *C* and *D*). Western blot indicated that Nrf2 expressions in drug-resistant cell lines were significantly higher than those in normal and sensitive cell line groups (Fig. 3, *E* and *F*). Consistently, RT–PCR results proved that Nrf2 expression was obviously increased in drug-resistant cells (Fig. 3*G*). To sum up, Nrf2 was highly denoted in AML drug–resistant cell lines.

### Overexpression of Nrf2 decreases the sensitivity of leukemia cells to Ara-C

**Figure 4:**
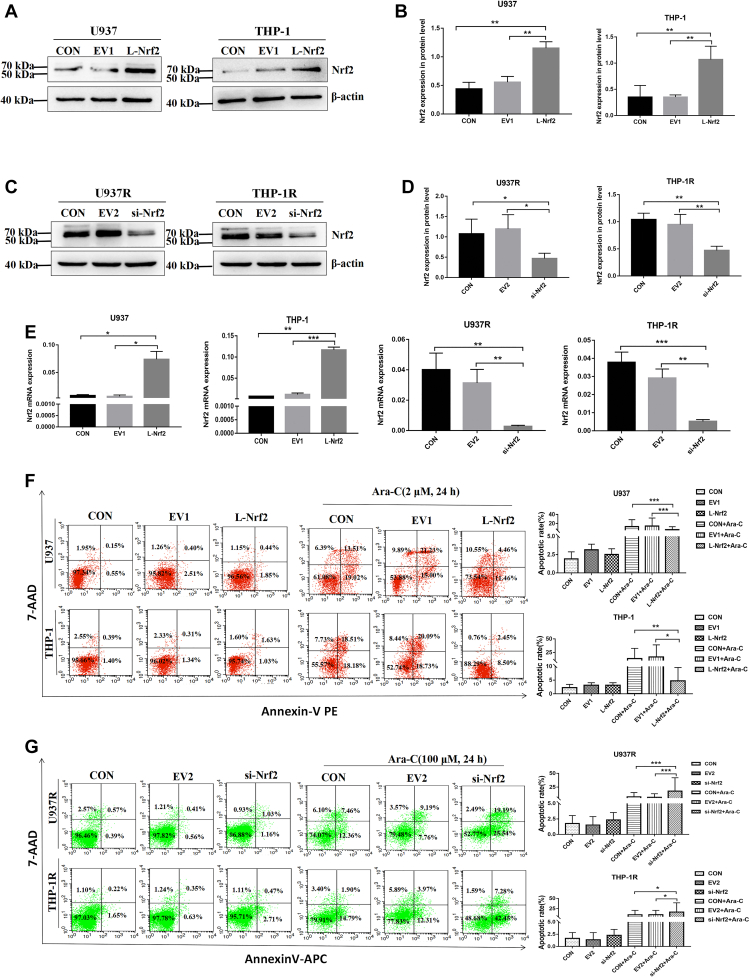
**High expression of Nrf2 decreases the sensitivity of leukemic cells to Ara-C.***A*, Western blot was used to verify the upregulation of Nrf2 in AML-sensitive cell lines (U937 and THP-1). *B*, the relative gray values of Nrf2 expression. *C*, Western blot was applied to detect the downregulation of Nrf2 in AML-resistant cell lines (U937R and THP-1R). *D*, the relative gray values of Nrf2 expression. *E*, RT–PCR verified the expression of mRNA after Nrf2 regulation. *F*, FCM was employed to detect the apoptosis rate of AML-sensitive cell lines cocultured with Ara-C (2 μM) for 24 h after Nrf2 overexpression. *G*, after downregulation of Nrf2, the apoptosis rate of AML drug–resistant cell lines cocultured with Ara-C (100 μM) for 24 h was detected by FCM. Each experiment was repeated three times independently and expressed as mean ± SD. ∗*p* < 0.05, ∗∗*p* < 0.01, and ∗∗∗*p* < 0.001. AML, acute myeloid leukemia; Ara-C, cytarabine; FCM, flow cytometry; Nrf2, nuclear factor E2–related factor 2.

Based on the aforementioned results, it could be speculated that the high expression of Nrf2 could be closely associated with drug resistance in AML cells, whereas its molecular mechanism remained unclear. So we upregulated Nrf2 in AML-sensitive cell lines and downregulated Nrf2 in AML-resistant cell lines. Western blot confirmed the expression of Nrf2 protein in sensitive cell lines (Fig. 4, *A* and *B*) and resistant cell lines (Fig. 4, *C* and *D*). RT–PCR was employed to detect the expression of Nrf2 mRNA in these cell lines (Fig. 4*E*). Subsequently, FCM was used to detect the effect of Nrf2 on the sensitivity of AML cell lines to Ara-C, indicating that compared with the control (CON) and empty vector 1 (EV1) groups, Nrf2 upregulation in U937 and THP-1 cell lines could significantly reduce the apoptotic number of leukemic cells (Fig. 4*F*). However, Nrf2 downregulation in U937R and THP-1R cells could enhance the sensitivity of drug-resistant cell lines to Ara-C (Fig. 4*G*). Therefore, Nrf2 overexpression could reduce the sensitivity of leukemia cells to Ara-C.

### Overexpression of Nrf2 promotes OGG1 expression to mediate drug resistance of AML cells to Ara-C

**Figure 5:**
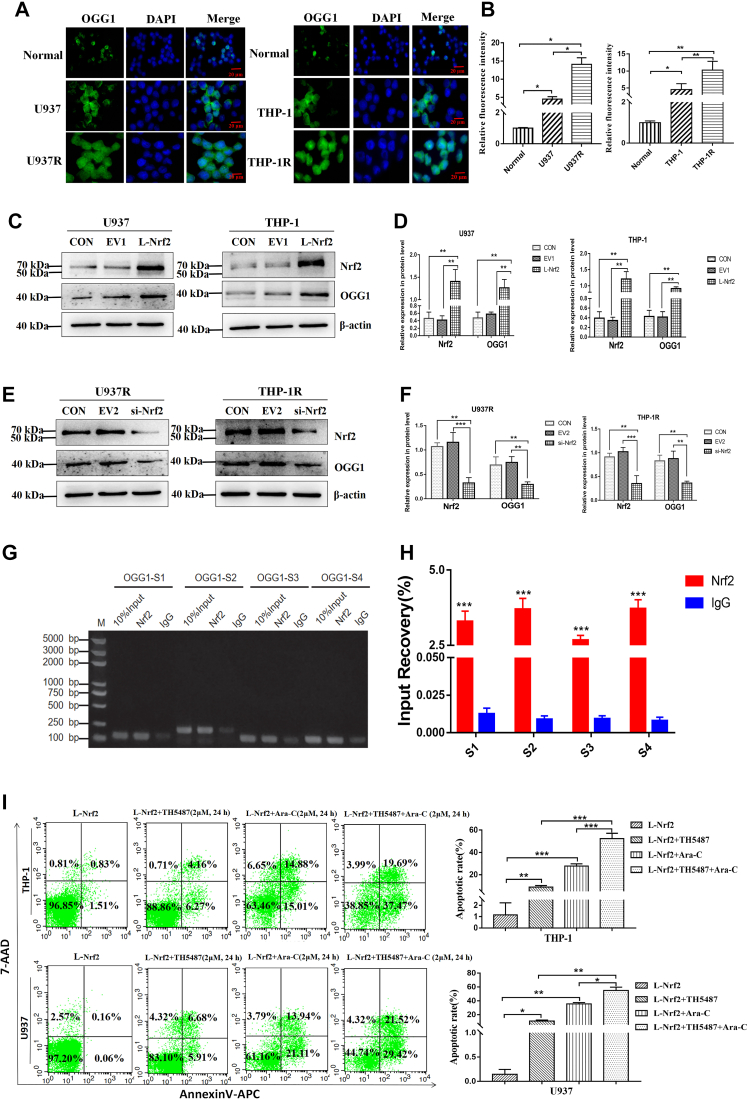
**Overexpression of Nrf2 promotes the expression of OGG1 to mediate drug resistance in AML cells.***A*, detection of OGG1 expression in normal (bone marrow mononuclear cells from normal healthy donors) and AML-sensitive cell lines (U937 and THP-1) and drug-resistant cell lines (U937R and THP-1R) by IF staining. Scale bars represent 20 μm. *B*, quantification of OGG1 relative fluorescence intensity in normal and leukemia cells by ImageJ. *C*, Western blot was employed to detect OGG1 protein expression after upregulation of Nrf2. *D*, the relative gray values of Nrf2 and OGG1 expressions. *E*, Western blot was adopted to identify the expression of OGG1 protein after downregulation of Nrf2. *F*, the relative gray values of Nrf2 and OGG1 expressions. *G*, analysis of DNA-enriched fragments of Nrf2 in THP-1R cells by agarose gel electrophoresis. *H*, ChIP–qPCR showed that the concentration of OGG1 promoter amplification products in anti-Nrf2 group was higher than that in anti-IgG group. S1: OGG1 promoter site 1; S2: OGG1 promoter site 2; S3: OGG1 promoter site 3; S4: OGG1 promoter site 4. IgG: negative control. 10% input: positive control. *I*, the apoptosis rate of AML cell lines upregulated by Nrf2 was detected by FCM after treatment with Ara-C (2 μM) combined with OGG1 inhibitor TH5487 (2 μM) for 24 h. Each experiment was repeated three times independently and expressed as mean ± SD. ∗*p* < 0.05, ∗∗*p* < 0.01, and ∗∗∗*p* < 0.001. AML, acute myeloid leukemia; Ara-C, cytarabine; ChIP–qPCR, chromatin immunoprecipitation–quantitative PCR; FCM, flow cytometry; IF, immunofluorescence; IgG, immunoglobulin G; Nrf2, nuclear factor E2–related factor 2; OGG1, 8-hydroxyguanine DNA glycosidase.

**Supplemental Fig. S1:**
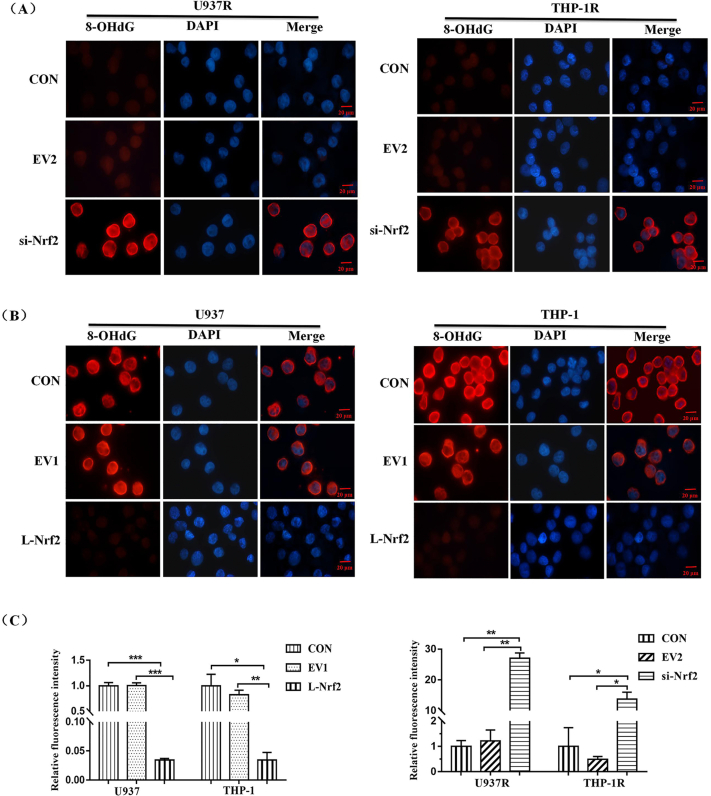
**The activity of OGG1 is higher in leukemia cells overexpressing Nrf2.***A* and *B*, immunofluorescence was used to detect the expression of 8-OHdG in leukemia cells. Scale bars = 20 μm. *C*, quantification of 8-OHdG relative fluorescence intensity in leukemia cells by Image J. Each experiment was repeated three times independently and expressed as mean ± SD. ∗*p* < 0.05, ∗∗*p* < 0.01, ∗∗∗*p* < 0.001.

Next, this work explored the possible mechanism by which Nrf2 influenced the biological characteristics of leukemic cells. In clinical samples, we found that there existed an obvious positive correlation between Nrf2 and BER pathway gene OGG1 expression. Immunofluorescence (IF) results demonstrated that the expression of OGG1 in drug-resistant cell lines was higher than that in normal groups and sensitive cell lines (Fig. 5, *A* and *B*). In addition, we also discovered that Nrf2 overexpression could efficiently promote the expression of OGG1 protein in U937 and THP-1 cells (Fig. 5, *C* and *D*). Meanwhile, the level of OGG1 protein decreased when Nrf2 was downregulated in AML drug–resistant cell lines (Fig. 5, *E* and *F*). The aforementioned results suggested that Nrf2 overexpression might promote the expression of OGG1 in AML cells. Furthermore, in order to evaluate the functional activity of OGG1, we adopted IF to detect the expression of 8-OHdG after regulating Nrf2. The results showed that after downregulation of Nrf2, the expression of 8-OHdG increased, indicating that the activity of OGG1 decreased (Fig. S1*A*). However, after upregulation of Nrf2, the expression of 8-OHdG decreased significantly, indicating that the activity of OGG1 increased (Fig. S1, *B* and *C*). To verify the interaction of Nrf2 with OGG1, we obtained the DNA fragment binding to transcription factor Nrf2 in THP-1R cells by chromatin immunoprecipitation (ChIP). The results of ChIP assay showed that Nrf2 specifically interacted with these binding sites in the OGG1 promoter region (Fig. 5*G*). Moreover, the existence of OGG1 promoter–binding sites in DNA fragment of THP-1R cells was verified by quantitative PCR (qPCR) experiment. The results showed that there were different degrees of binding between Nrf2 and the four binding sites of OGG1 promoter region in THP-1R cells (Fig. 5*H*). Subsequently, to further explore whether inhibition of OGG1 could enhance the sensitivity of AML cells to Ara-C, we used OGG1 inhibitor TH5487 combined with Ara-C to detect the apoptotic number in AML cell lines U937 and THP-1 after upregulating Nrf2. Compared with the group treated with Ara-C or TH5487 alone, the apoptotic number of AML cells subject to treatment with Ara-C combined with TH5487 increased conspicuously (Fig. 5*I*). Therefore, Nrf2 might mediate the resistance of AML cells to Ara-C by regulating the expression of OGG1.

### Downregulation of OGG1 increased the sensitivity of AML drug–resistant cell lines to Ara-C

**Figure 6:**
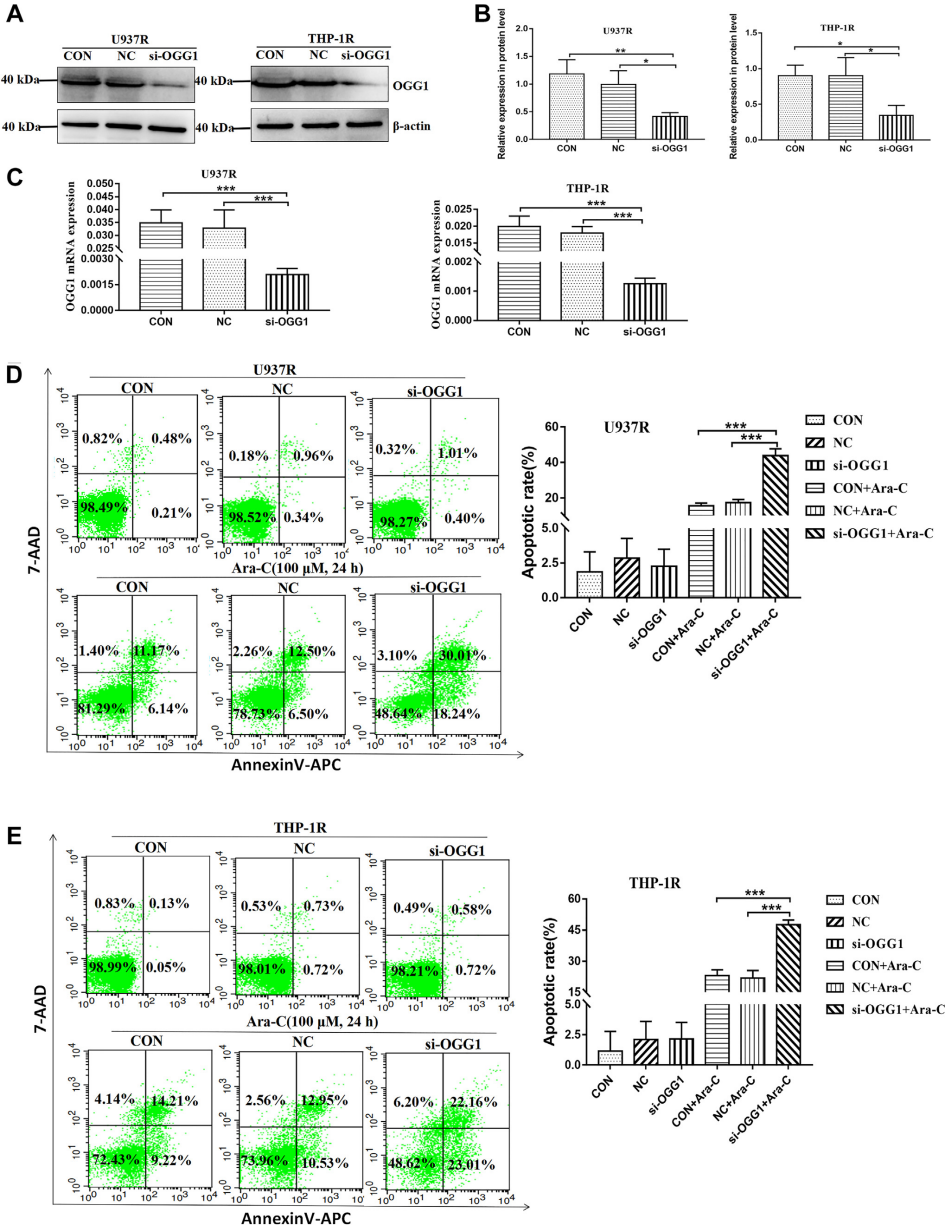
**Downregulation of OGG1 increased the sensitivity of AML-resistant cells to Ara-C.***A*, Western blot was applied to detect the protein expression of OGG1 after transfection with OGG1 siRNA (si-OGG1) for 72 h in AML-resistant cell lines (U937R and THP-1R). *B*, the relative gray values of OGG1 expression. *C*, RT–PCR verified the mRNA expression of OGG1 after transfection with si-OGG1 for 72 h in AML-resistant cell lines (U937R and THP-1R). *D* and *E*, after transfection with si-OGG1 for 72 h, the apoptosis rate of AML drug–resistant cell lines cocultured with Ara-C (100 μM) for 24 h was detected by FCM. Each experiment was repeated three times independently and expressed as mean ± SD. ∗*p* < 0.05, ∗∗*p* < 0.01, and ∗∗∗*p* < 0.001. AML, acute myeloid leukemia; Ara-C, cytarabine; FCM, flow cytometry; OGG1, 8-hydroxyguanine DNA glycosidase.

Furthermore, we explored the effect of OGG1 expression on AML drug–resistant cell lines. AML drug–resistant cell lines (U937R and THP-1R) were transfected with OGG1 siRNA (si-OGG1) for 72 h. The transfection effect of si-OGG1 was verified by Western blot and RT–PCR. The results showed that the expression levels of OGG1 protein and mRNA were significantly lower than those in CON and negative control (NC) groups (Fig. 6, *A*–*C*). Furthermore, the AML drug–resistant cell lines were transfected with si-OGG1 for 72 h and then cocultured with 100 μM Ara-C for 24 h. The results of FCM indicated that the sensitivity of AML drug–resistant cell lines to Ara-C increased significantly after downregulation of OGG1 (Fig. 6, *D* and *E*). These results suggested that OGG1 plays an important role in drug resistance of AML.

### Overexpression of Nrf2 promotes the expression of OGG1 by activating AKT signaling pathway in AML

**Figure 7:**
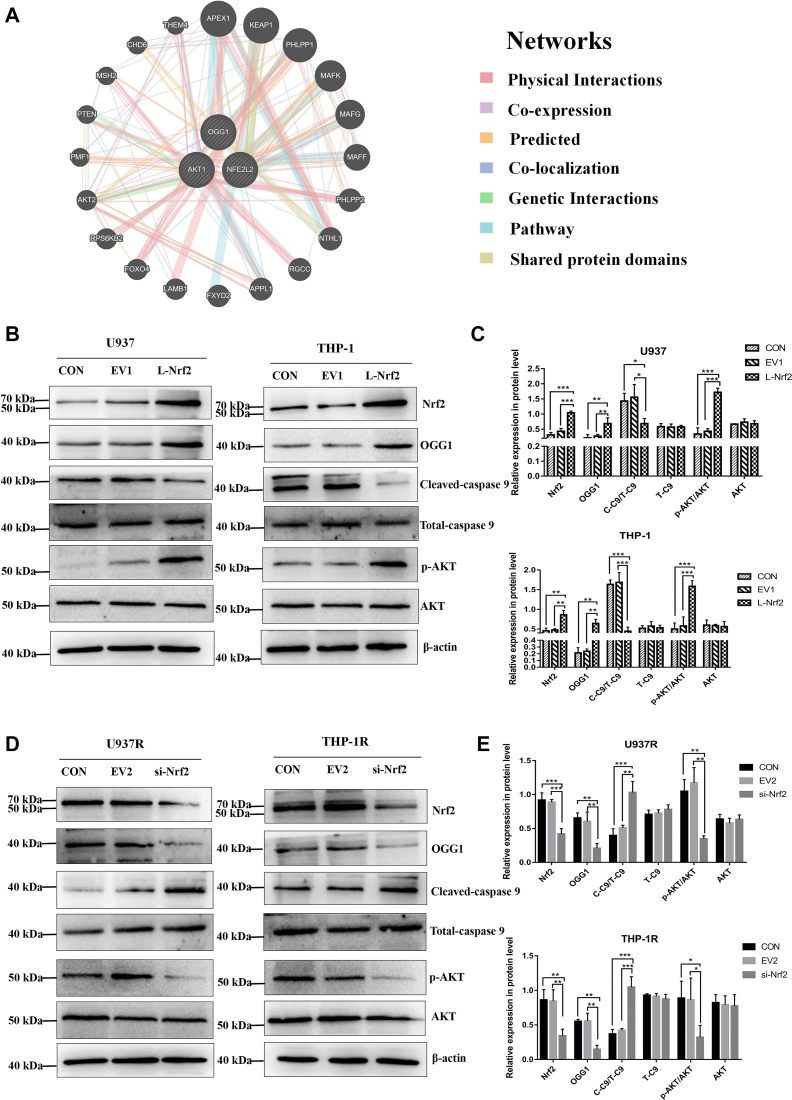
**Nrf2 overexpression promotes OGG1 expression by activating AKT signaling pathway.***A*, GeneMania protein–protein interaction network of OGG1, Nrf2, and AKT. *B* and *C*, Western blot was applied to identify the expressions of Nrf2, OGG1, AKT, p-AKT, Cleaved-caspase 9, and Total-caspase 9 proteins in AML-sensitive cell lines after overexpression of Nrf2. *D* and *E*, Western blot was adopted for detecting the expression of Nrf2, OGG1, AKT, p-AKT, Cleaved-caspase 9, and Total-caspase 9 proteins in AML drug–resistant cell lines after downregulation of Nrf2. Each experiment was repeated three times independently and denoted as mean ± SD. ∗*p* < 0.05, ∗∗*p* < 0.01, and ∗∗∗*p* < 0.001. Nrf2, nuclear factor E2–related factor 2; OGG1, 8-hydroxyguanine DNA glycosidase.

Based on the aforementioned results, we further explored the possible molecular mechanism that Nrf2 overexpression promoted OGG1 expression and mediated AML drug resistance. Previous study reported that luteolin inhibits oxidative damage in ARPE-19 cells by promoting the expression of Nrf2 and activating AKT signaling pathway (29). Inhibition of PI3K/AKT signaling pathway can further inhibit the process of glycolysis and then interfere with ATP production, finally inducing apoptosis of AML cells (30). Based on this study, AKT is considered to play an essential role in the progression of AML. To study the potential mechanism of Nrf2 regulating OGG1, we applied GeneMania protein–protein interaction network to analyze the relationship between Nrf2 and OGG1. The results revealed that Nrf2 might regulate OGG1 through AKT signaling pathway (Fig. 7*A*). On this basis, we assessed the effect of regulating Nrf2 on AKT signaling pathway using Western blot. The results proved that the level of phosphorylated AKT (p-AKT) was significantly higher in upregulation of Nrf2 group than that in CON and EV1 groups for U937 and THP-1 cell lines, whereas the expression of Cleaved-caspase 9 apoptotic protein decreased (Fig. 7, *B* and *C*). Meanwhile, the expression of p-AKT was detected in U937R and THP-1R groups with downregulation of Nrf2. The results also revealed that the expression level of p-AKT in downregulated Nrf2 groups was lower than that in CON and EV2 groups, whereas the expression of Cleaved-caspase 9 increased (Fig. 7, *D* and *E*). The aforementioned findings suggested that overexpression of Nrf2 could activate the transduction of AKT signaling pathway.

**Figure 8:**
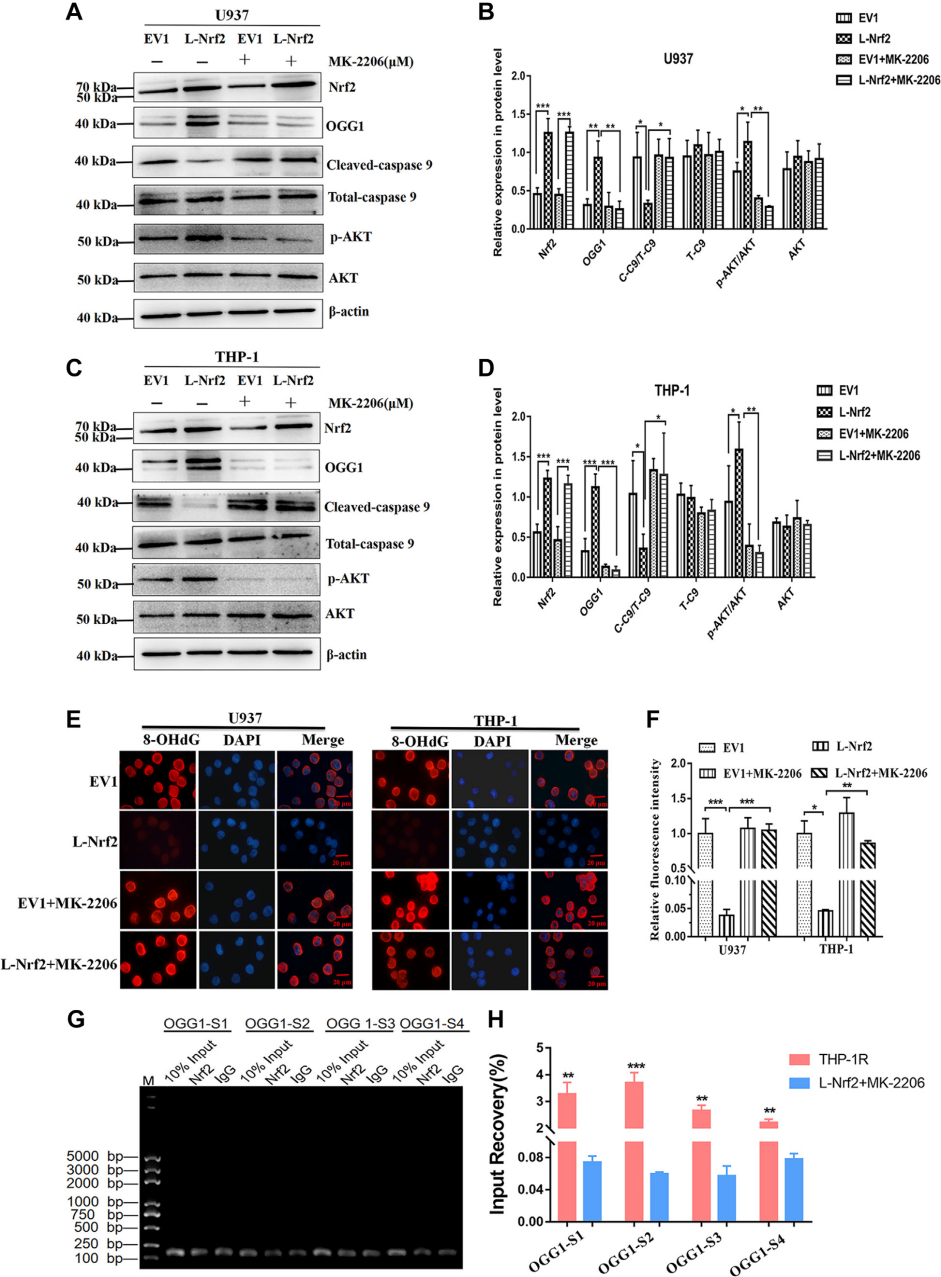
**Inhibition of AKT signaling pathway to reduce OGG1 expression.***A*, after U937 cells were treated with 2 μM MK-2206 for 24 h, the protein expression levels of Nrf2, OGG1, AKT, p-AKT, Cleaved-caspase 9, and Total-caspase 9 in Nrf2 overexpression and EV1 groups were detected by Western blot. *B*, the relative gray value of related proteins. *C*, after THP-1 cells were treated with 2 μM MK-2206 for 24 h, the protein expression levels of Nrf2, OGG1, AKT, p-AKT, Cleaved-caspase 9, and Total-caspase 9 in Nrf2 overexpression and EV1 groups were detected by Western blot. *D*, the relative gray values of related proteins. *E*, after U937 and THP-1 cells were treated with 2 μM MK-2206 for 24 h, the expression levels of 8-OHdG in Nrf2 overexpression and EV1 groups were detected by IF. Scale bars represent 20 μm. *F*, quantification of 8-OHdG relative fluorescence intensity in leukemia cells by ImageJ. *G*, after coculture of THP-1 cells overexpressing Nrf2 with 2 μM MK-2206 for 24 h, analysis of DNA-enriched fragments of Nrf2 in THP-1 cells by agarose gel electrophoresis. IgG: negative control. 10% input: positive control. S1: site 1; S2: site 2; S3: site 3; and S4: site 4. *H*, ChIP–qPCR showed that the concentration of OGG1 promoter amplification products in THP-1R group was higher than that in THP-1 cells with overexpression of Nrf2 treated with MK-2206. Each experiment was repeated three times independently and expressed as mean ± SD. ∗*p* < 0.05, ∗∗*p* < 0.01, and ∗∗∗*p* < 0.001. ChIP–qPCR, chromatin immunoprecipitation–quantitative PCR; EV1, empty vector 1; IF, immunoflorescence; IgG, immunoglobulin G; Nrf2, nuclear factor E2–related factor 2; OGG1, 8-hydroxyguanine DNA glycosidase; 8-OHdG, 8-hydroxy-2-deoxyguanosine.

We further verified whether Nrf2 promoted the expression of OGG1 by activating AKT signaling pathway. U937 and THP-1 cells overexpressing Nrf2 were treated with 2 μM MK-2206 (AKT signaling pathway inhibitor) for 24 h. There existed no obvious difference in Nrf2 expression in leukemia cells treated with MK-2206, but the protein levels of p-AKT and OGG1 decreased significantly, whereas Cleaved-caspase 9 increased significantly (Fig. 8, *A*–*D*). In addition, U937 and THP-1 cells overexpressing Nrf2 were treated with 2 μM MK-2206, and the expression of 8-OHdG was detected by IF. The results showed that the expression of 8-OHdG decreased in the upregulated group of Nrf2, indicating that the activity of OGG1 increased. However, after coculture with MK-2206 for 24 h, the expression of 8-OHdG was higher, indicating that the activity of OGG1 decreased (Fig. 8, *E* and *F*). Next, in order to verify that Nrf2 regulates OGG1 through AKT signaling pathway to mediate drug resistance in leukemia cells, we verified the interaction between Nrf2 and OGG1 after inhibition of AKT signaling pathway by ChIP. Agarose gel electrophoresis showed that the binding of Nrf2 and OGG1 decreased after the THP-1 cells overexpressing Nrf2 were cocultured with 2 μM MK-2206 for 24 h (Fig. 8*G*). ChIP–qPCR showed that the four binding sites of Nrf2 and OGG1 decreased significantly after treatment with MK-2206 (Fig. 8*H*). Therefore, overexpression of Nrf2 could promote the expression of OGG1 and mediate drug resistance in AML cells by activating AKT signaling.

### AML cells with low Nrf2 expression have a lower risk of drug resistance *in vivo*

**Figure 9:**
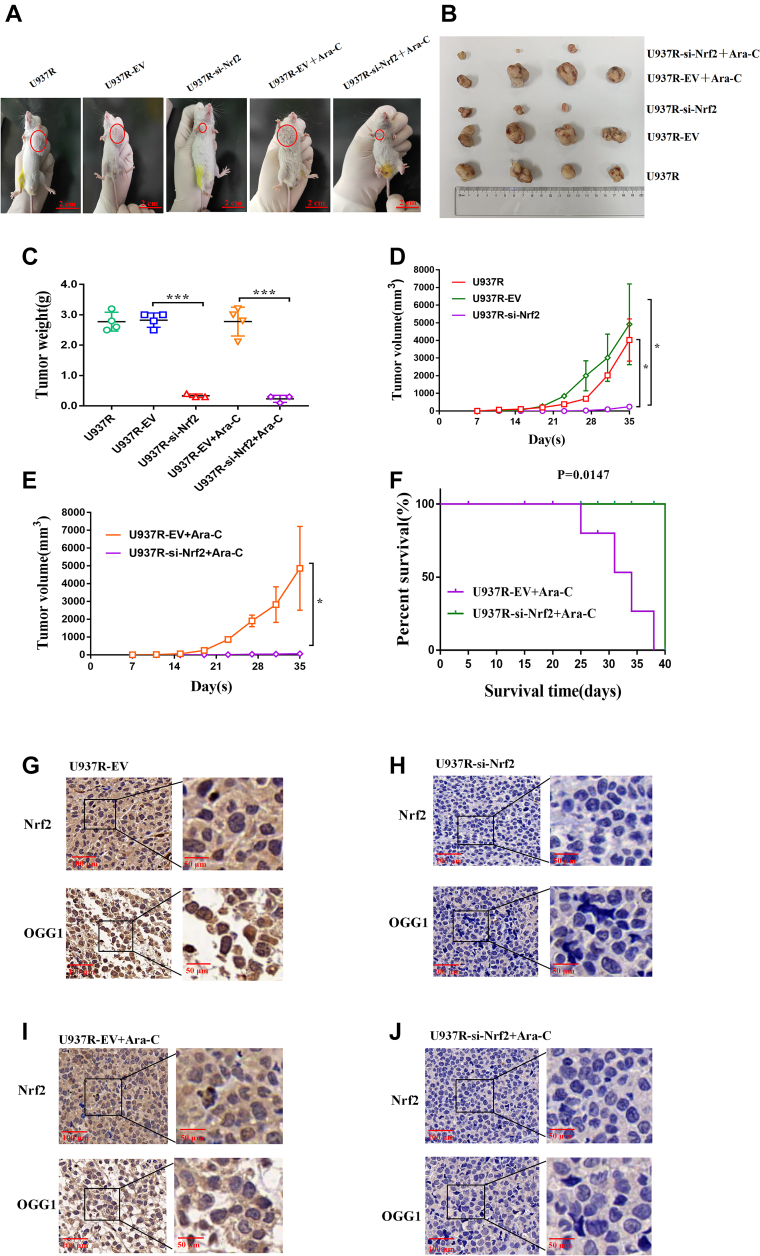
**Downregulation of Nrf2 reduces the risk of drug resistance *in vivo*.***A* and *B*, tumor volume of mice after subcutaneous injection of U937R (n = 4), U937R-EV (n = 4), U937R-si-Nrf2 (n = 3), and U937R-EV + Ara-C (n = 4) and U937R-si-Nrf2 + Ara-C (n = 3) cells after 35 days growth. *C*, changes of tumor weight after subcutaneous injection of U937R (n = 4), U937R-EV (n = 4), U937R-si-Nrf2 (n = 3), and U937R-EV + Ara-C (n = 4) and U937R-si-Nrf2+Ara-C (n = 3) cells. *D* and *E*, volume growth curve of xenograft tumor. *F*, analysis and drawing of survival curve of xenografted tumor mice by Kaplan–Meier method. *G*–*J*, IHC was applied to identify the expressions of Nrf2 and OGG1 in subcutaneous transplanted tumor tissue sections. The scale bars from *left* to *right* represent 100 and 50 μm, respectively. ∗*p* < 0.05, ∗∗*p* < 0.01, and ∗∗∗*p* < 0.001. Ara-C, cytarabine; EV, empty vector; IHC, immunohistochemistry; Nrf2, nuclear factor E2–related factor 2; OGG1, 8-hydroxyguanine DNA glycosidase.

The xenotransplantation model was established to evaluate the effects of Nrf2 on the growth of leukemic cells *in vivo*. Nonobese diabetic/severe combined immunodeficiency (NOD/SCID) mice were randomly categorized into U937R group, U937R-EV group, U937R-si-Nrf2 (silenced Nrf2) group, U937R-EV + Ara-C group, and U937R-si-Nrf2 + Ara-C group. When the tumor was palpable, the mice were exposed to the treatment with Ara-C immediately. The results showed that compared with U937R and EV groups, downregulated Nrf2 tumors were smaller in volume (Fig. 9, *A* and *B*) and lighter in weight (Fig. 9*C*). Moreover, in comparison with U937R and EV groups, the tumor in the si-Nrf2 group grew more slowly (Fig. 9*D*). The tumor growth in si-Nrf2 group after Ara-C treatment was slower than that in EV + Ara-C group (Fig. 9*E*). As presented in Figure 9*F*, the survival time of mice transplanted with si-Nrf2 cells was the longest after Ara-C treatment, whereas the total survival time of mice transplanted with EV cells was shorter. Moreover, the expressions of Nrf2 and OGG1 in tumor tissues were identified by immunohistochemistry (IHC) assay. Compared with the EV group, the expression of OGG1 decreased after si-Nrf2 (Fig. 9, *G* and *H*). Meanwhile, the expression of OGG1 in si-Nrf2 group was lower than that in EV + Ara-C group after Ara-C treatment (Fig. 9, *I* and *J*). These data showed that low expression of Nrf2 reduced the expression of OGG1 and inhibited the tumor growth, thus exerting a certain protective effect on xenografted mice.

## Discussion

Reversing drug resistance remains a major challenge in tumor chemotherapy. Ara-C combined with Nrf2 inhibitors can increase the sensitivity of AML cells to chemotherapeutic drugs (16). Increased Nrf2 expression is tightly associated with the chemotherapy resistance of multiple tumors (31, 32, 33). BER is the core of DDR mechanism (34). The overexpression of BER pathway makes it resistant to temozolomide in malignant primary brain tumors (9). Therefore, inhibition of enzymes in BER pathway may generate certain antitumor effect. In AML study, patients with high expression of OGG1 have poor relapse-free survival (26). Furthermore, OGG1 is an efficient target for T lymphocytic leukemia cells (35). Compared with AML cells with high OGG1 expression, deficient cells were more sensitive to Ara-C (36). In addition, OGG1 gene polymorphism can also increase the probability of relapse in patients undergoing AML (37). The obtained evidence indicates that Nrf2 and OGG1 play a momentous role in AML relapse and drug resistance, but its mechanism remains unclear. For the first time, we confirmed that overexpression of Nrf2 mediated the role of OGG1 expression in drug resistance of AML. In GEPIA dataset, the expressions of Nrf2 and OGG1 in AML samples were higher than those in normal samples, which were validated by our clinical samples and drug-resistant cell lines, implying that Nrf2 and OGG1 might be potential drug resistance genes in AML.

To further explore the relationship between Nrf2 and OGG1, we upregulated the expression of Nrf2 in sensitive AML cell lines and downregulated Nrf2 in drug-resistant AML cell lines. It is suggested that Nrf2 could positively regulate the expression of OGG1. In addition, we also confirmed that the activity of OGG1 increased and the expression of 8-OHdG decreased after upregulation of Nrf2. Meanwhile, the results revealed that the apoptotic number of upregulated Nrf2 cells significantly decreased, whereas apoptosis significantly increased after Nrf2 downregulation. These findings indicated that Nrf2 overexpression reduces the sensitivity of AML cells to chemotherapeutic drug Ara-C. Accumulating evidence has revealed that Nrf2 activation promotes its binding to the OGG1 promoter region, thus exerting a protective role in cells (28, 38). However, it remains unclear whether there exists a binding between Nrf2 and OGG1 in leukemia cells. So we applied ChIP experiment to verify that OGG1 promoter contained the binding region of Nrf2, suggesting that overexpressed Nrf2 could enhance OGG1 expression by directly binding to its promoter region.

Previous studies show that OGG1 inhibitors can reduce the interaction between OGG1 and DNA, thereby preventing the repair effect of OGG1 on damaged cells (39, 40). Based on the aforementioned literature reports and our previous research results, it could be demonstrated that OGG1 might be the key factor of drug resistance in AML cells. In this study, we confirmed that the apoptotic number of leukemic cells treated with OGG1 inhibitor TH5487 combined with Ara-C increased significantly. Meanwhile, we found that downregulation of OGG1 could significantly increase the sensitivity of AML drug–resistant cells to Ara-C. These results proved that OGG1 inhibition could reduce the drug resistance of AML cells.

We continued to explore the potential molecular mechanism of Nrf2 promoting OGG1 expression and mediating drug resistance in AML cells. Celastrol can promote the expression of Nrf2 and activate PI3K/AKT signaling, thus lowering oxidative stress level (41). In addition, many studies have revealed that AKT signaling pathway generates a vital role in AML. For example, exo-miR-7-5p derived from BM mesenchymal stem cells promotes AML cell apoptosis by blocking the phosphorylation of PI3K/AKT/mammalian target of rapamycin signaling (42). Hematopoietic kinase inhibitors combined with 5-azacytidine or Ara-C can decrease AKT/extracellular signal–regulated kinase phosphorylation and increase the expressions of apoptotic proteins (43). Moreover, some studies have demonstrated that the continuous activation of PI3K/AKT signaling may be the cause of AML drug resistance (30). However, it remains unclear whether Nrf2 regulates the expression of OGG1 and mediates AML resistance through AKT signaling pathway. Therefore, this study further explored the effects of Nrf2, OGG1, and AKT signaling on leukemia cells. The results demonstrated that overexpression of Nrf2 decreased the apoptotic proteins by activating AKT signaling. After treated with AKT pathway inhibitor MK-2206, it was found that the activity of OGG1 decreased and the expression of 8-OHdG increased. To further investigate whether the inhibition of AKT pathway could affect the function of Nrf2–OGG1 axis, through ChIP experiment, it was found that the combination of Nrf2 and OGG1 decreased after the inhibition of AKT signaling pathway, which hindered the function of Nrf2–OGG1 axis. It is suggested that the activation of AKT pathway could increase the binding of Nrf2 and OGG1, which mediates the drug resistance of AML. Previous studies have also shown that the activation of AKT signaling pathway can induce the increase of OGG1 expression (44, 45). However, it has been reported that reactive oxygen species induced by high glucose activates AKT pathway to downregulate the expression of OGG1 (46). The reasons for this discrepancy have not yet been revealed. Our results showed that activation of AKT signaling pathway could increase the expression of OGG1, which is consistent with the results of the former study.

Through *in vivo* experiments, we further confirmed that Nrf2 downregulation could inhibit OGG1 expression and generate a certain protective effect on NOD/SCID mice. However, this kind of research has not been reported. Because of the limitation of experimental conditions, whether the combination of OGG1 inhibitor with Ara-C could inhibit the growth of AML cells and prolong the survival time of mice in the case of high expression of Nrf2 has not been confirmed. Therefore, we need to further explore in the follow-up research.

To conclude, this work showed for the first time that Nrf2 overexpression promotes OGG1 expression by activating AKT signaling, which mediates drug resistance of AML cells to Ara-C. Inhibition of AKT signaling pathway reduces the expression of OGG1, which hinders the function of Nrf2–OGG1 axis. Furthermore, downregulation of OGG1 could increase the sensitivity of leukemia cells to Ara-C. Hence, in the future, novel OGG1 inhibitors should be developed in combination with standard chemotherapeutic drugs in the treatment of malignant hematological tumors. This might be an important discovery of AML drug resistance and could provide a novel breakthrough for clinical overcoming leukemia drug resistance.

## Experimental procedures

### Collection of clinical samples

According to the Chinese guidelines for diagnosis and treatment of AML (nonacute promyelocytic leukemia) and relapsed/refractory AML, patients with complete remission and relapse were included in the study. Acute promyelocytic leukemia is a special subtype of AML, its treatment is different from other types of AML, and patients can get better remission rate and long-term survival after treatment (47); it is not included in this study. Using the method of random sampling, 81 BM samples of AML patients (including relapse [n = 46], complete remission [n = 35]) in the Affiliated Hospital of Guizhou Medical University from July 2020 to October 2021 were collected. Normal donors (normal healthy donors, n = 28) were used as control for the isolation of BM monocytes from AML patients and normal healthy donors by Ficoll density centrifugation for follow-up experiments. We have obtained the approval of the institutional ethics committee and the right of informed consent from the patients in advance. Table 1 provides characteristics of AML patients.

**Table 1:** Characteristics of patient samples

Patients no.	Age (years)	Gender	FAB subtype	WBC (10^9^/l)	HB (g/l)	PLT (10^9^/l)	BM blast (%)	Relapse/complete remission
1	71	M	M5	5.16	74.00	33.00	48.83	Relapse
2	49	F	M4	28.87	68.00	51.00	87.11	Relapse
3	33	M	M2	10.28	113.00	78.00	40.78	Relapse
4	61	F	M4	17.85	109.00	12.00	20.63	Relapse
5	57	F	M4	0.86	66.00	29.00	11.52	Relapse
6	49	M	M5	2.26	63.00	97.00	27.18	Relapse
7	32	M	M2	21.70	59.00	18.00	12.31	Relapse
8	29	M	M4	51.75	114.00	9.00	86.01	Relapse
9	43	F	M4	4.24	102.00	293.00	18.53	Relapse
10	52	M	M5	46.88	60.00	72.00	29.17	Relapse
11	43	F	M5	4.24	102.00	293.00	18.53	Relapse
12	31	M	M2	7.74	61.00	35.00	62.74	Relapse
13	55	M	M2	0.45	62.00	22.00	42.83	Relapse
14	87	M	M5	3.84	77.00	15.00	59.95	Relapse
15	33	M	M2	3.73	102.00	67.00	40.78	Relapse
16	57	F	M2	7.89	94.00	277.00	15.51	Relapse
17	40	F	M4	131.98	89.00	23.00	75.15	Relapse
18	48	F	M2	69.15	85.00	68.00	84.88	Relapse
19	34	M	M2	0.13	63.00	6.00	53.28	Relapse
20	30	F	M5	10.43	64.00	41.00	76.81	Relapse
21	28	F	M4	37.48	64.00	5.00	28.00	Relapse
22	25	F	M2	1.73	98.00	21.00	9.74	Relapse
23	55	M	M2	112.64	67.00	70.00	9.84	Relapse
24	40	M	M5	36.21	76.00	27.00	76.34	Relapse
25	43	F	M5	10.37	90.00	39.00	40.04	Relapse
26	61	F	M5	27.40	63.00	12.00	44.00	Relapse
27	55	F	M4	6.78	50.00	21.00	30.09	Relapse
28	42	F	M5	9.75	91.00	372.00	15.51	Relapse
29	73	M	M2	17.27	120.00	13.00	88.91	Relapse
30	63	F	M2	3.01	97.00	474.00	7.19	Relapse
31	35	M	M4	2.37	74.00	44.00	49.67	Relapse
32	33	M	M2	8.21	39.00	5.00	12.31	Relapse
33	24	F	M5	81.53	63.00	24.00	87.03	Relapse
34	57	M	M4	3.95	71.00	8.00	73.95	Relapse
35	52	F	M5	2.99	69.00	24.00	43.85	Relapse
36	29	F	M4	21.45	93.00	101.00	65.67	Relapse
37	39	M	M5	2.10	73.00	44.00	17.09	Relapse
38	56	F	M4	6.09	70.00	115.00	17.56	Relapse
39	63	F	M4	6.86	58.00	70.00	45.29	Relapse
40	65	M	M4	0.60	60.00	32.00	7.35	Relapse
41	34	M	M2	3.24	73.00	44.00	52.13	Relapse
42	19	M	M5	17.58	63.00	7.00	43.14	Relapse
43	25	F	M2	1.66	98.00	18.00	9.74	Relapse
44	41	M	M4	48.56	79.00	49.00	88.39	Relapse
45	73	F	M2	14.39	72.00	29.00	52.93	Relapse
46	41	M	M4	48.22	77.00	59.00	88.44	Relapse
47	35	F	M6	4.05	85.00	312.00	2.08	Complete remission
48	59	M	M4	2.62	62.00	29.00	0.50	Complete remission
49	48	F	M2	5.27	77.00	280.00	0.81	Complete remission
50	23	F	M2	5.84	115.00	262.00	0.72	Complete remission
51	21	M	M4	2.36	93.00	178.00	1.78	Complete remission
52	73	M	M4	4.84	129.00	138.00	0.49	Complete remission
53	51	F	M2	11.58	116.00	267.00	1.22	Complete remission
54	42	F	M5	5.09	107.00	208.00	1.05	Complete remission
55	47	F	M5	3.83	72.00	169.00	1.00	Complete remission
56	55	F	M2	2.00	61.00	39.00	1.21	Complete remission
57	50	F	M1	2.69	106.00	61.00	3.57	Complete remission
58	33	M	M2	10.30	83.00	357.00	1.23	Complete remission
59	50	M	M2	3.06	70.00	76.00	1.01	Complete remission
60	22	M	M2	10.15	66.00	213.00	0.65	Complete remission
61	21	M	M4	6.29	91.00	195.00	1.89	Complete remission
62	38	M	M5	4.26	81.00	142.00	1.87	Complete remission
63	47	F	M2	4.22	135.00	162.00	0.20	Complete remission
64	38	M	M2	5.99	137.00	167.00	1.76	Complete remission
65	34	F	M2	2.73	94.00	117.00	3.68	Complete remission
66	58	M	M2	5.12	105.00	258.00	0.21	Complete remission
67	36	M	M2	5.01	130.00	144.00	1.39	Complete remission
68	51	M	M2	5.62	98.00	136.00	0.65	Complete remission
69	48	F	M1	4.90	61.00	660.00	1.65	Complete remission
70	51	M	M4	15.01	112.00	207.00	0.96	Complete remission
71	49	F	M4	5.60	98.00	245.00	0.23	Complete remission
72	23	F	M2	8.66	125.00	362.00	1.46	Complete remission
73	63	F	M2	6.79	129.00	203.00	0.46	Complete remission
74	24	M	M4	3.78	92.00	282.00	2.71	Complete remission
75	46	F	M4	6.88	98.00	168.00	0.83	Complete remission
76	48	F	M4	1.80	97.00	230.00	0.94	Complete remission
77	16	M	M4	1.40	125.00	91.00	1.34	Complete remission
78	53	F	M2	5.78	107.00	153.00	0.52	Complete remission
79	50	F	M1	5.64	108.00	155.00	0.79	Complete remission
80	18	M	M1	7.24	155.00	122.00	1.31	Complete remission
81	38	M	M5	7.11	100.00	341.00	0.50	Complete remission

Abbreviations: F, female; FAB, French–American–British; HB, hemoglobin; M, male; PLT, platelet; WBC, white blood cell.

### Cell culture

Human leukemic cell lines THP-1 and U937 were provided by Guizhou Hematopoietic Stem Cell Transplantation Center Laboratory. The cells were cultivated in RPMI1640 medium (10% fetal bovine serum) at 37 °C and 5% CO_2_ incubator. In addition, 1% penicillin (100 unit/ml) and streptomycin (100 mg/ml) were supplemented to the culture medium. Meanwhile, drug-resistant cell lines THP-1R and U937R were established by increasing Ara-C concentration gradient. When the cells proliferated to normal shape, the impact of the aforementioned drugs was repeated. Each concentration was applied for 3 to 5 times. The drug concentration was added gradually. The drug induction lasted 6 to 8 months until the cells could grow stably in the final concentration.

### RT–PCR

Total RNA was extracted by RNA kit, and then RNA was reverse transcribed into complementary DNA by reverse transcription kit (Tiangen Biotech). Next, the sample complementary DNA was tested by RT–PCR primer (Generay Biotech) and SYBR Green kit (Tiangen Biotech). The cycle threshold (CT) values of the samples were detected by Bio-Rad instrument. Using β-actin as the reference, the relative expression of the target gene was computed by comparative CT values (2^−ΔCT^). The RT–PCR primer sequence is provided in Table 2.

**Table 2:** The characteristics of the primers adopted for RT–PCR

Gene	Sequence (5′->3′)	Sequence (5′->3′)
β-actin	Forward primer	CTACCTCATGAAGATCCTCACCGA
	Reverse primer	TTCTCCTTAATGTCACGCACGATT
APE1	Forward primer	CAATACTGGTCAGCTCCTTCG
	Reverse primer	TGCCGTAAGAAACTTTGAGTGG
XRCC1	Forward primer	CCTTTGGCTTGAGTTTTGTACG
	Reverse primer	CCTCCTTCACACGGAACTGG
Nrf2	Forward primer	TTCCCGGTCACATCGAGAG
	Reverse primer	TCCTGTTGCATACCGTCTAAATC
POL-β	Forward primer	TGTTACATCAGGTTGTGGAGCAGTTAC
	Reverse primer	ACCCATGAACTTTGTCTCACCCTTTG

### Reagents and antibodies

Ara-C, TH5487 (OGG1 inhibitor), and MK-2206 (AKT inhibitor) were purchased from MCE. The Nrf2 antibody was acquired from the abcam, and the OGG1 antibody was purchased from the Novus. AKT/p-AKT and 8-OHdG antibodies were brought from Santa Cruz Biotechnology. Cleaved-caspase 9 and Total-caspase 9 antibodies were purchased from Immunoway. β-actin antibody was provided by Proteintech.

### Western blot

AML cells (clinical samples and cell lines) were lysed with radioimmunoprecipitation assay lysate containing 1% PMSF, and the concentration of protein was determined by bicinchoninic acid kit. Protein (30 μg) was loaded on 10% SDS-PAGE gel and transferred to polyvinylidene difluoride membranes. After blocking with sealing solution (5% skim milk) at chamber temperature for 2 h, the membranes were subsequently incubated with target antibody (primary antibody) at 4 °C overnight. The dilution ratio was 1:1000 for Nrf2, OGG1, AKT, p-AKT, Cleaved-caspase 9, and Total-caspase 9 antibodies. The next day, the secondary antibody was nurtured at chamber temperature for 1 h. The membranes were then washed with Tris-buffered saline with Tween-20. The expression of target protein was detected by electrochemiluminescence luminescence kit. Finally, with β-actin protein as the internal reference, the gray values of the obtained bands were analyzed by ImageJ (National Institutes of Health) software.

### Staining procedure of ICC, IF, and IHC

We first fixed the cells with 4% paraformaldehyde at chamber temperature for 2 h. After PBS rinsing, the cells were fixed on adhesive slides and dried at 37 °C. Triton X-100 (0.1%) was used for 30 min cell permeation, followed by PBS washing for three times. ICC and IF experiments were then carried out. ICC was carried out using antigen repair solution to repair 5 min of cells at higher temperature. Then the adhesive slides were placed in a wet box and incubated with goat serum for 1 h. After PBS washing, Nrf2 antibody (1:50 dilution) and OGG1 antibody (1:20 dilution) were diluted with fluorescent antibody diluent and incubated overnight at 4 °C. The cells were rinsed for three times and raised with fluorescent secondary antibody (1:100 dilution) for 1 h. Diaminobenzidine and hematoxylin staining were used for 5 min and 30 s in turn. After washing, gradient dehydration was performed in 50%, 75%, 95%, and 100% ethanol. After placed in xylene for 5 min permeation, the slides were dried at room temperature. In IF, the adhesive slides were placed in a wet box and incubated with goat serum for 1 h. Following treatment with OGG1 (1:20 dilution) and 8-OHdG (1:50 dilution) antibody, the slides were then incubated with fluorescent secondary antibody (1:100 dilution) for 1 h. After PBS washing, the nucleus was stained for 5 min with 4′,6-diamidino-2-phenylindole. Finally, the fluorescence image was captured by fluorescence microscope. In IHC, the expressions of Nrf2 and OGG1 in tumor tissues were detected using IHC assay, and the dilution ratios of primary antibody and secondary antibody were 1:20 and 1:100, respectively.

### FCM

After treatment with different concentrations of Ara-C for 24 h, AML cells were collected and the number of apoptosis was identified. PE Annexin V apoptosis detection kit and Annexin V-APC/7-AAD apoptosis kit was adopted to detect the number of apoptosis in cells transfected with lentivirus according to the instructions. For the cells not transfected with lentivirus, AnnexinV-FITC and propidium iodide (7Sea Pharmatech Co, Ltd) were used for 15 and 5 min staining in turn. FCM was adopted for detecting and analyzing the number of apoptosis.

### ChIP

Leukemia cells were collected and fixed with 1% paraformaldehyde. The ChIP experiment was carried out according to the instructions of EZ-Magna ChIP A/G ChIP Kit (Merck Millipore). Each bottle of cells was incubated with 10× glycine of 1 ml at room temperature for 5 min to stop the cross-linking reaction and collect nuclei. The DNA was broken by ultrasound, and the DNA fragments were mainly enriched between 300 and 500 bp. Next, the agarose gel electrophoresis experiment was carried out. The antibodies and cellular ultrasonic chromatin were incubated overnight at 4 °C. The dosage of Nrf2 antibody was 4 μg. Immunoglobulin G was used as NC. Eluted protein–DNA complex, uncrosslinked and purified DNA. Next, the PerfectStart Green qPCR SuperMix (Transgene) kit was used to detect the binding of Nrf2 and OGG1 promoter regions. The characteristics of the OGG1 promoter primers are provided in Table 3.

**Table 3:** The characteristics of the OGG1 promoter primers

Gene	Site	Primers	Sequence (5′-3′)
OGG1 promoter	S1	F	TCTGTGCCCCAGGGATGATA
		R	CTGCCACTCACTCCATGCAT
	S2	F	CTGAACTGCCAGGGGAAGAG
		R	AAGAAGGTGCTGAGGTTGGG
	S3	F	ACTGAGACAATGATGGCACTGG
		R	GACTGCAAATTCTTGAAGAGCAACA
	S4	F	ACAGCAACCCCAAATCCCTAT
		R	TCTAGTCGCCTGGAGTAGGAG

Abbreviations: F, forward; R, reverse.

### Cell transfection

According to the lentivirus transfection kit (Genechem), U937, THP-1, U937R, and THP-1R cells were transfected with lentivirus particles (overexpressed Nrf2, L-Nrf2) and human Nrf2-RNAi (si-Nrf2), respectively. U937, THP-1, U937R, and THP-1R cells transfected with EV were applied as controls. After amplification and maintenance in RPMI1640 medium for 7 days, U937/U937R and THP-1/THP-1R cell lines stably denoting L-Nrf2 and si-Nrf2 were screened with 1 and 2 μg/ml puromycin, separately. The targeting si-OGG1 sequence and NC siRNA were provided by RiboBio Co, Ltd. The NC siRNA has no significant sequence similarity to mouse, rat, or human gene sequences. Si-OGG1-Forward: 5′-ACACUGGAGUGGUGUACUAdTdT-3′ and si-OGG1-Reverse: 5′-UAGUACACCACUCCAGUGUdTdT-3′. According to the manufacturer's instructions, the drug-resistant cell lines were transfected with si-OGG1, and NC was used. After stable transfection of si-OGG1 for 72 h, it was verified by Western blot and RT–PCR.

### Cell Counting Kit-8 assay

The sensitivity of leukemic cell lines to Ara-C was detected by Cell Counting Kit-8 method. The cells with a density of 3 × 10^4^ per 100 μl were evenly added to 96-well plate, with five repetitive holes in each group. After exposed to different concentrations of drugs for 24 h, Cell Counting Kit-8 reagent (10 μl) was supplemented to each well. After coculture for 1 to 2 h, the absorbance was computed by microplate spectrophotometer at 450 nm. The IC_50_ was calculated using GraphPad Prism 7.0 (GraphPad Software, Inc), followed by assessing drug resistance ratio of AML drug–resistant cell lines.

### Xenograft tumor model

Approved by the Experimental Animal Ethics Committee of Guizhou Medical University, the xenograft tumor model was carried out in 4- to 6-week-old NOD/SCID male mice (Sibef). NOD/SCID mice were subcutaneously injected with 1 × 10^7^ cells/200 μl, U937R, U937R-EV, U937R-si-Nrf2 cells, respectively. When the tumor tissue was palpable, 60 mg/kg/d Ara-C was injected intraperitoneally into mice for 7 days. The tumor length (L) and width (W) were computed every other day, and the tumor volume was calculated according to the formula: tumor volume = 0.5 ∗ L ∗ W^2^. The growth of mice was observed every 2 days. After the mice in the chemotherapy group died of natural causes, the mice in the nondeath group were sacrificed under anesthesia, and the tumors were collected.

### Statistical analysis

Data were explored by SPSS (IBM) 19.0 software and plotted by GraphPad Prism 7.0 software. Independent-sample *t* test was employed to perform the analysis between two groups, and single factor analysis of variance was applied for multiple groups. The experimental data are denoted as mean ± SD. The meaning of *p* value is as follows: ∗*p* < 0.05, ∗∗*p* < 0.01, and ∗∗∗*p* < 0.001. Among them, *p* < 0.05 is considered to be statistically significant.

## Data availability

All the data produced for this work are contained within the article.

## Supporting information

This article contains supporting information.

## Conflict of interest

The authors declare that they have no conflicts of interest with the contents of this article.
